# Ethical issues in pain and omics research. Some points to start the debate

**DOI:** 10.3325/cmj.2014.55.1

**Published:** 2014-02

**Authors:** Christian Compagnone, Fernanda Tagliaferri, Massimo Allegri, Guido Fanelli

**Affiliations:** 1 Department of Anesthesia, Intensive Care and Pain Therapy, University Hospital of Parma, Parma, Italy; 2 Department of Clinic Surgical Pediatric and Diagnostic Sciences, Pain Therapy Service, University of Pavia-Fondazione IRCCS Policlinico San Matteo, Pavia, Italy

Pain is still a major public health problem, with a high prevalence of both acute and chronic conditions. The inclusion of genomic (and other omics) technologies represents a new approach in pain research. However, new research and diagnostic tools in the field of health care always require a detailed study of ethical aspects and implications. In particular, pain research represents an area with many challenges when it comes to ethical aspects. Using placebo treatment, an agent biologically inert in respect to pain condition but potentially helpful for the patient, represents a classic paradigmatic type of ethical concerns in this area (1,2). Placebo treatment is based on the concept of deception. It undermines honest relationship and trust between doctor and patient, which is extremely important for a successful treatment (1). Still, application of placebo in terms of analgesia will cause a positive response in 35% of patients (3). It is important to highlight that response to placebo does not mean that the patient is faking the pain and, therefore, it would be unethical to withhold the specific treatment. Functional magnetic resonance imaging of the brain showed that placebo analgesia produces activation of relevant brain areas (4). On the other hand, intentionally giving placebo treatment for a condition that can be adequately treated would be against the right of patients to receive the best possible care.

Another controversial subject are patients unable to “correctly” express pain or to give consent. In animal research, important improvements are made in order to avoid suffering and pain of the involved animals (5). But what happens when the main issue of research is pain? Animal research is intended to be limited to those studies where the animal model is the only possibility. A controversial editorial comment in Nature Neuroscience, with the provocative title “Italian biomedical research under fire,” pointed out the risks of excessive restrictions in animal research (6). Although there is an overall agreement to avoid “avoidable” pain, the question remains what is “avoidable.” A particular issue is fetal, neonatal, and infant pain. In 1985, the infant Jeffrey Lawson underwent open heart surgery awake and conscious throughout the entire procedure. The opinion of the anesthesiologist was that it had never been demonstrated that premature babies felt pain (7). In contrast, recently a study protocol including the use of placebo control to study neonatal pain in an intensive care unit has not been approved by the institutional review board (8). However, fetal surgeries continue to be performed with little (or no) regard about pain. Practical problems such as correct monitoring and physiological issues (immaturity of the central nervous system) can be difficult to address even in a standard approach. Pain research in cognitively impaired people is of particular interest because the prevalence of both conditions increases with age. There has been considerable evidence on inadequate assessment and treatment of pain in this vulnerable population. It seems that unfortunately research that supports best practices for assessing and treating pain in cognitively impaired patients is limited by methodological obstacles (9). In the current practice of clinical research, this population is excluded from studies. The development of new monitoring tools for animal and human pain could be an innovative path to resolve these problems. The desired result would be to maintain the minimum intensity of pain required to achieve the goals of the study and to hold this level of intensity during its entire course.

Another important point in pain research is the lack of knowledge about pain treatment by the general population, and in particular by health care personnel (10). Physicians and nurses involved in pain treatment showed lack of preparation regarding pain (11) and patients had misconceptions about pain treatment. Famous examples are that the use of opioids always produces addiction and that “pain is part of the cure.” Some countries, like Italy, modified the law to include mandatory pain monitoring in the clinical records (12). At the moment, several medical associations are studying the inclusion of pain assessment and treatment skills in the resident study programs.

Inclusion of genomic (and other omics) technologies in pain research represents a delicate topic and it raises several questions and concerns. Genomic (and other omics) technologies in the last two decades have produced not only a deep change in biomedical research, but also generated a change in the agenda of institutional review boards and research ethics. The potential to discover large amounts of possibly important incidental information during the genomic analysis has been identified as one of the greatest challenges to genomic medicine (13). Debates should be focused on the physicians’ obligations arising from this type of the research, protection of the included individuals, and communication of such research to patients and the community (14). Omics analyses in humans raise complex ethical issues, which often significantly impact the pace of research programs. By working collaboratively, researchers intend to answer questions never addressed before, but at the same time different team members may have varied ethical perceptions due to differences in disciplines as well as legal traditions and cultures. Furthermore, recent studies have demonstrated how “easy” it is to deduce individual identity from diverse public data sets (Genomes Project Consortium Nature 2012) (15). The authors were able to establish the identity of close to 50 of the Center for Study of Human Polymorphisms participants (both women and men). It is important to note that the authors did not reveal the names of the participants (or violated any known policies), but merely demonstrated their ability to identify them through the analysis of publicly available information. These findings alert us about how easy information is available in the era of information networks. Particular attention should be paid to preserve the privacy right in this new global village (16).

In conclusion, innovative tools in pain research could introduce new answers to old questions. However, there are numerous ethical issues to consider. Ethical pain research should not only guarantee the right of patients to the correct treatment, but also it should assure the inclusion of excluded categories of patients (fetal, pediatric, and cognitively impaired patients) with particular solutions for specific queries. The use of innovative models and monitoring would be useful in this setting. Omics research adds an additional variable to the equation. It provides important benefits to research, but increases the vulnerability of those affected when patients’ privacy is concerned.
